# A real-world observational study on the effect of Qingre Lishi decoction on glycemic profile using continuous glucose monitoring in obese type 2 diabetes adults

**DOI:** 10.3389/fendo.2024.1372593

**Published:** 2024-07-23

**Authors:** Bingchen Wei, Tianshu Gao, Mingzhe Li, Xiaojun Tian, Jinxi Wang

**Affiliations:** ^1^ The First Clinical College, Liaoning University of Traditional Chinese Medicine, Shenyang, China; ^2^ Endocrinology Department, the First Affiliated Hospital of Liaoning University of Traditional Chinese Medicine, Shenyang, China; ^3^ Internal Medicine Department, the Third Affiliated Hospital of Liaoning University of Traditional Chinese Medicine, Shenyang, China; ^4^ The Fourth Encephalopathy Department, Shenyang Second Hospital of Traditional Chinese Medicine, Shenyang, China; ^5^ Teaching Laboratory Center, Liaoning University of Traditional Chinese Medicine, Shenyang, China

**Keywords:** newly diagnosed type 2 diabetes mellitus, type of damp-heat trapped spleen, Qingre Lishi decoction, CGM, blood glucose fluctuation

## Abstract

**Objective:**

To observe the clinical efficacy and safety of the Qingre Lishi decoction in treating of newly diagnosed overweight and obese patients with type 2 diabetes mellitus (T2DM) from an evidence-based medical perspective.

**Methods:**

70 cases of overweight and obese patients with newly diagnosed T2DM treated in the outpatient clinic of the Department of Endocrinology of the Affiliated Hospital of Liaoning University of Traditional Chinese Medicine from December 2021 to November 2022 were selected, of which 35 cases were in the observation group and 35 cases were in the control group. The observation group was treated with the Qingre Lishi decoction add lifestyle intervention, and the control group was treated with lifestyle intervention only. We compared and analyzed the fasting blood glucose (FPG), 2-hour postprandial glucose (2hPG), the occurrence of adverse reactions, and the related indexes provided by wearing the CGM device during the observation period of the patients in the two groups.

**Results:**

53 participants completed the clinical trial. In relation of glycemic control, a decreasing trend has shown in both groups, with the decreases in FPG, 2hPG, eHbA1c, and MG in the observation group being higher than those in the control group (*P*<0.05). In regard to blood glucose attainment, at the 28d, the attainment rate of patients in the observation group with TIR>80% was 87.10%, and the magnitude of changes in the rise of TIR and the fall of TAR was significantly better than that in the control group (*P*<0.01). In terms of blood glucose fluctuation, CV and SD of the patients in the observation group decreased compared with the 0d; the magnitude of daytime blood glucose fluctuation was significantly alleviated compared with that of the control group. The degree of decrease in LAGE, MAGE, and MODD was significantly lower than that of the control group (*P*<0.01).

**Conclusion:**

The Qingre Lishi decoction can effectively improve the hyperglycemic condition of overweight and obese patients with newly diagnosed T2DM. It can reduce blood glucose, alleviate blood glucose fluctuations, reduce the incidence of hypoglycemia, and improve patients’ adherence and self-confidence in controlling blood glucose.

**Clinical Trial Registration:**

https://itmctr.ccebtcm.org.cn/, identifier ITMCTR2024000006.

## 1 Introduction

The number of newly diagnosed type 2 diabetes (T2DM) patients in the world is increasing year by year and growing faster than before, of which the proportion of overweight people has reached 41.0%, while the proportion of obese people is 24.3% (1). Additionally, only 30.2% of those with a body mass index (BMI) over 28 have achieved glycated hemoglobin (HbA1c) control (2, 3). In 2021, the definition and criteria for alleviating T2DM were clearly stated at the American Diabetes Association (ADA) (4). Subsequently, the *Consensus of Chinese Experts on the Remission of Type 2 Diabetes Mellitus* was officially promulgated, re-emphasizing the importance of alleviating T2DM (5). Varies of domestic and international studies have confirmed that early intensive lifestyle intervention and medication for overweight and obese T2DM patients can substantially improve their hyperglycemic state and delay the progression of T2DM (6–8). Therefore, we have committed to alleviating T2DM as the ultimate goal of treating patients with newly diagnosed T2DM.

Nowadays, an increasing number of patients with newly diagnosed T2DM in China are opting to use Chinese herbs to manage their blood glucose levels. The *Guideline for the Prevention and Treatment of Type 2 diabetes mellitus in China* (2020 edition), along with several meta-analyses and randomized controlled studies, confirm that early herbal treatment for newly diagnosed T2DM can reduce patients’ symptoms, effectively regulate their blood glucose. In addition to this, herbal medicine can improve the β-cell functional index and insulin sensitivity post-treatment while restoring their own pancreatic islet function (9–16). For instance, Chinese herbal ginseng and astragalus compounds can rectify the instability of the internal environment caused by pathogenic factors and relieve inflammation, subsequently reducing blood glucose and improving the patients’ quality of life.

After years of clinical experience, our research team has concluded that the pathogenic perspective of Damp-heat induced Wasting-thirst (17, 18). We believe that most newly diagnosed T2DM are overweight, and mainly belongs to the type of Damp-heat trapped spleen, with severe insulin resistance and pancreatic islets β-cells damage. From multiple perspectives, including animal experiments and clinical evidence-based researches, we found that in overweight or obese T2DM individuals, those belonging to the type of Damp-heat trapped spleen showed significantly elevated levels of clear IL-6 and PRA, Ang II, and ALD of the RASS system (19–21). In this way, it was confirmed that impaired glucose regulation mechanisms contribute to the exacerbation of oxidative stress in vascular endothelium. Combined with the contemporary high-sugar and high-fat dietary pattern, we also found that pancreatic β-cells in individuals with T2DM often experience overload, resulting in repetitive stimulation of the vascular endothelium and subsequent development of oxidative stress, which then leads to persistent fluctuations in blood glucose (22–24). Similarly, an increasing number of experimental studies focusing on glucose-lipid metabolism, intestinal flora, and other aspects of overweight/obesity T2DM have shown common characteristics related to Damp-heat trapping the spleen. These studies have also revealed a disordered inflammatory regulatory mechanism in the body, dysfunction in adipokines and intestinal flora, as well as damaged or dysfunctional pancreatic islet β-cells (25–30). Although numerous evidence-based studies have been conducted to showcase the efficacy of Chinese herbal medicines in reducing and controlling blood glucose, the majority of these studies have combined with treatment of both traditional Chinese medicine and Western medicine.

However, this approach fails to provide a comprehensive evaluation of the actual effectiveness of Chinese herbal medicines alone. In a word, the principle of blood glucose lowered by traditional Chinese medicine still lacks the basis of clinical observation. Therefore, from the perspective of evidence-based medicine, our team treated newly diagnosed overweight and obese T2DM patients with the addition and subtraction of the Qingre Lishi decoction and evaluated its clinical efficacy and safety. Additionally, we integrated a continuous glucose monitoring system and a mobile application device to evaluate the impacts of treatment with the addition and subtraction of the Qingre Lishi decoction on blood glucose fluctuations.

## 2 Methods

### 2.1 Study design and participants

In this trial, 70 overweight and obese patients with newly diagnosed T2DM in the Department of Endocrinology of the Affiliated Hospital of Liaoning University of Traditional Chinese Medicine between December 2021 and November 2022 were selected. All participants were included according to the T2DM criteria defined in the ADA *Guidelines for the Diagnosis and Treatment of Diabetes* (2021 edition) and *Guideline for the Prevention and Treatment of Type 2 diabetes mellitus in China* (2020 edition). This research was approved by Ethics Committee of the First Affiliated Hospital of Liaoning University of Traditional Chinese Medicine [Y2023109CS(KT)-109-01]. And it was also registered with the code of ITMCTR2024000006 (Registration date: 15/01/2024) in the International Traditional Medicine Clinical Trial Registry.

#### 2.1.1 Specific inclusion criteria

a. newly diagnosed T2DM, with no comorbidities or complications of diabetes; b. the course of the disease is within 12 months (including 12 months); c. the age of patients were between 18 and 65, regardless of gender; d. overweight and obese, BMI ≥ 24kg/m^2^, defined by Chinese criteria (30); e. no COVID-19 infections in the last 6 months, 48 hours negative for novel coronavirus-N gene test and negative for novel coronavirus-ORF1ab gene test.

#### 2.1.2 The exclusion criteria

a. failure to meet the new diagnosis of T2DM; b. women who are pregnant or breastfeeding; c. those with severe heart, lung, brain, liver and kidney diseases; d. combination of any diabetic comorbidities and complications of diabetes mellitus; e. allergy or intolerance to therapeutic drugs; f. severe mental disorders, functional neurologic disorders and inability to communicate properly; g. other diseases that may have an effect on glucose metabolism; h. experience of a critical illness or other stressful situation within the last month; i. participation in other studies within the last 3 months; j. COVID-19 infection in the last 6 months, or 48 hours positive for novel coronavirus-N gene and positive for novel coronavirus-ORF1ab gene. Termination criteria: Newly diagnosed overweight and obese T2DM remained HbA1c/eHbA1c > 7.5% after 1 month of treatment with the addition and subtraction of the Qingre Lishi decoction.

#### 2.1.3 Procedure

All 70 eligible participants underwent an OGTT test upon enrollment and wore ambulatory glucose monitoring. At the same time, the attending physician provided them with training and guidance on the application of CGM and APP software, and conducted follow-up visits at the 14d and 28d after enrollment. Each participant was required to wear the CGM throughout the observation period, including during showering and sleeping. The participants were provided with dietary instructions for managing diabetes, specifying a consumption of 30 kcal/(kg·d), distributed across three meals, with 50% from carbohydrates, 15% from protein and 35% from fat (9). The exercise instruction requires each patient to walk slowly for 30 minutes each morning. Smoking, alcoholic beverages, or drinks containing alcohol are not allowed during the observation period.

The observation group was given Traditional Chinese Medicine, the Qingre Lishi decoction. Essential medicine composition: radix bupleuri (Chai Hu, 柴胡) 15g, rhizoma pinellinae praeparata (Fa Banxia, 法半夏) 15g, scutellaria baicalensis (Huang Qin, 黄芩) 15g, wine-treated rhubarb (Jiu Dahuang, 酒大黄) 15g, sinocalamus affinis (Zhu Ru, 竹茹) 15g, fructus aurantii immaturus rhizome (Zhi Shi, 枳实) 10g, anemarrhenae (Zhi Mu, 知母) 10g, raw gypsum (Sheng Shigao, 生石膏) 15g, coptis chinensis (Huang Lian, 黄连) 15g, cassia twig (Gui Zhi, 桂枝) 10g, rhizoma zingiberis (Gan Jiang, 干姜) 10g, dark plum (Wu Mei, 乌梅) 5g, schisandra chinensis (Wu Weizi, 五味子) 5g. Method of administration: prepare 300 mL of the above Chinese medicine after decocting it in water, and take 100 mL of it warm during the three meals in a day, analyze the pattern of disease in combination with the participants, and adjust the treatment accordingly. All participants in the control group expressed a voluntary preference for lifestyle interventions over medication. At the same time, they were willing to receive CGM to better understand their glycemic changes. Apart from that the control group remained consistent with the observation group in meeting all other requirements.

### 2.2 Outcomes and measures

#### 2.2.1 Primary outcome

The primary was the change from baseline levels in relevant glycemic indicators at the 14d and 28d. Indicators include fasting blood glucose (FPG, mmol/L) and 2-hour postprandial blood glucose (2hPG, mmol/L) obtained after the OGTT test; estimated HbA1c (eHbA1c, %), standard deviation (SD, mmol/L), mean amplitude of glycemic excursions (MAGE, mmol/L), large amplitude of glycemic excursions (LAGE, mmol/L), mean of daily differences (MODD, mmol/L), coefficient of variation (CV, %), time in range (TIR, %): percentage of time with blood glucose between 3.9 and 10.0 mmol/L, time above range (TAR, %): percentage of time with blood glucose ≥10.0 mmol/L, time below range (TBR, %), mean blood glucose (MG, mmol/L) in CGM reports.

#### 2.2.2 Secondary outcomes

Included BMI, triglyceride (TG, mmol/L), low density lipoprotein (LDL-C, mmol/L), alanine aminotransferase (ALT, U/L), aspartate aminotransferase (AST, U/L), γ-glutamyl transpeptidase (GGT, U/L), serum creatinine (Scr, μmol/L), urea nitrogen (UREA, mmol/L).

#### 2.2.3 Laboratory data measures

In this study, FPG, TG, LDL-C, ALT, AST, GGT, Scr, and UREA were measured with a fully automated biochemical analyzer (HITACHI Model 7600-020; Hitachi, Japan; Immunoturbidimetric method). Normal reference range for monitoring biochemical indicators: FPG: 3.9 ~ 6.1mmol/L, TG: 0.7 ~ 1.7mmol/L, LDL-C: ≤ 3.62mmol/L, ALT: 5 ~ 40U/L, AST: 8 ~ 40U/L, GGT: 11 ~ 50U/L, Scr: 59 ~ 104μmol/L, UREA: 2.9 ~ 8.2mmol/L. Detection of HbA1c and FCP by fully automated electrochemiluminescence immunoassay analyzer (Cobas Model e601; Roche, Germany; immunoluminescent method). Normal reference range for monitoring biochemical indicators: HbA1c: 4.0% ~ 6.0%, FCP: 1.1 ~ 4.4ng/mL. The ATTD International Consensus recommends a TIR attainment cutoff of 70 percent. Considering that this study is on newly diagnosed T2DM, the higher the percentage of TIR attainment, the more it contributes to their T2DM remission, so 80% was chosen as the cut-off point for TIR attainment in this study (31).

#### 2.2.4 Glucose measures

The CGM monitor was used in this study to monitor patients’ daily blood glucose in real time. The continuous glucose monitoring system (model: GS1, Registration Certificate No.: National Equipment Standard 20213070871) produced by Shenzhen Silicon-Based Sensing Technology Co., Ltd. is selected for blood glucose monitoring, including the sensor package and the mobile phone Silicon-based Dynamic APP software (version: 01.11.00.00). The sensor package includes the sensor electrode assembly and guide pin, while the applicator includes the transmitter and transmitter back glue. The effective range of the blood glucose test for this instrument is 2.2 ~ 25 mmol/L.

The sensing probe is inserted into the subcutaneous tissue of the inner side of the participant’s upper arm. It receives an electrical signal each minute, storing and recording a factual blood glucose value each 5 minutes. This amounts to 288 values per day, providing continuous monitoring of the participant’s blood glucose for 14 days, resulting in a total of 4021 blood glucose values. If the participant’s blood glucose value exceeds the effective range, the mobile app will display an alert message stating very low blood glucose or very high blood glucose accompanied by an alarm sound. After wearing the device for 14 days, the APP system automatically generates an AGP map based on the recorded values.

### 2.3 Statistical analysis

Data processing was performed with SPSS 25.0 statistical software. All measures conforming to a normal distribution expressed by means ± standard deviation (SD), and non-normally distributed measures were expressed by medians (interquartile range), except for separate labeling. Independent *t* test and paired *t* test were used to analyze and compare between and within groups for data conforming to normal distribution, respectively, while non-normally distributed data were compared between and paired within groups using non-parametric rank sum test and Friedman test. *P<* 0.05 was considered a statistically significant difference, and *P<* 0.01 was considered a statistically significant difference.

## 3 Results

**Figure 1:**
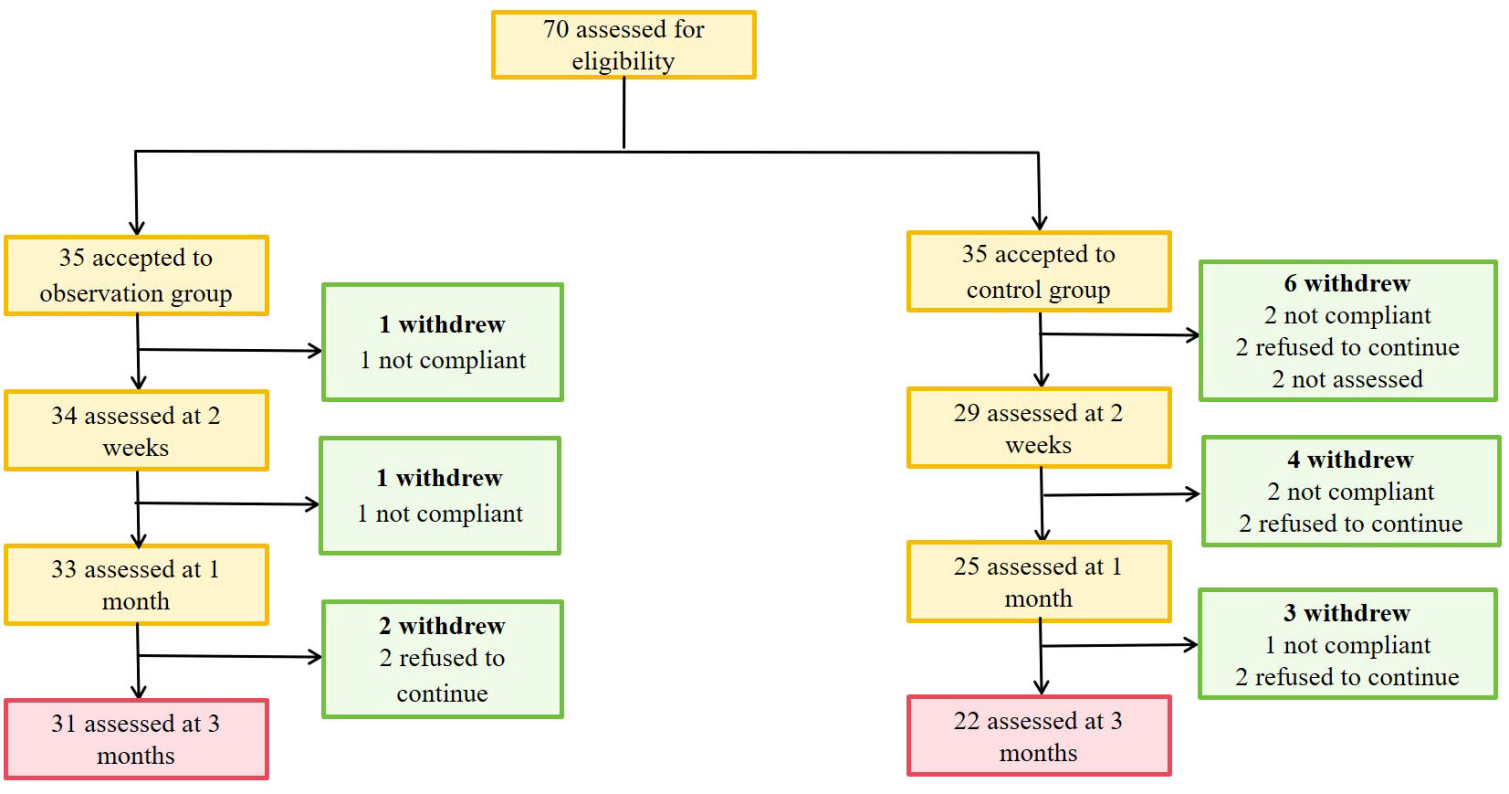
Progress of the research.

A total of 53 patients finally completed the observation. Among the dropouts, there were 4 cases in the observation group where 2 cases refused to continue with the original medication or clinical regimen, and 2 cases exhibited poor adherence during the wearing process. In the control group, there were 13 dropouts where 5 cases could not be revisited for various reasons, 2 cases showed poor adherence during the wearing process, and 6 cases refused to continue with the original medication or the clinical treatment regimen, and asked to withdraw from the study (**Figure 1**). There were 26 males and 27 females, with an average age of (47.36 ± 11.71) years, BMI (27.17 ± 2.39) kg/m^2^, and FPG (venous blood) 9.42 ± 2.44 mmol/L. During the observation process, the differences in age, disease duration, BMI and blood glucose indexes between the two groups were not statistically significant (*P* > 0.05) (**Tables 1**, **2**).

**Table 1:** Comparison of baseline characteristics between the observation and control groups [ $\bar{X\;}$ ± s or M (Ql, Qu)].

Parameters	Observation group	Control group	*P* value
Gender (*n*)			
Male	16	10	0.659
Female	15	12	
Age (years)	45.77 ± 11.36	49.59 ± 12.09	0.246
Course of disease (months)	1.50 (0.50,12.00)	1.00 (0.50,4.00)	0.439
BMI (kg/m^2^)	27.00 ± 2.34	27.40 ± 2.49	0.549
Education (0~12years/>13years)			
0~12	12	13	0.143
>13	19	9	
Physical activity intensity (*n*)			
Low	14	12	0.822
Moderate	12	7	
High	4	3	
Smoking (*n*)			
Yes	7	2	0.197
No	24	20	
Drinking (*n*)			
Yes	9	6	0.889
No	22	14	
Family history of diabetes Genetic history (*n*)			
Yes	8	8	0.409
No	23	14	

Body mass index (BMI).

**Table 2:** Comparison of basic biochemical indexes between observation and control groups [ $\bar{X\;}$ ± s or M (Ql, Qu)].

Parameters	Observation group	Control group	*P* value
C peptide (ng/mL)	3.20 (2.33,4.98)	2.91 (1.97,3.29)	0.159
LDL-C (mmol/L)	2.83 ± 1.32	2.91 ± 0.99	0.805
TG (mmol/L)	1.92 (1.25,3.25)	1.94 (1.07,2.68)	0.718
ALT (U/L)	27.74 ± 13.21	29.86 ± 13.89	0.579
AST (U/L)	25.06 ± 11.33	32.18 ± 11.92	0.034
GGT (U/L)	24.84 ± 9.75	35.55 ± 11.99	0.001
Scr (μmol/L)	61.71 ± 13.49	58.77 ± 12.85	0.426
UREA (mmol/L)	5.60 (3.80,7.30)	5.79 ± 1.28	0.752

Low density lipoprotein (LDL-C), triglyceride (TG), alanine aminotransferase (ALT), aspartate aminotransferase (AST), γ-glutamyl transpeptidase (GGT), serum creatinine (Scr), urea nitrogen (UREA).

### 3.1 Comparison of changes in blood glucose control indexes before and after observation

Throughout the trial, we noticed a decrease in FPG, 2hPG, and eHbA1c levels in newly diagnosed overweight and obese T2DM patients when compared to the 0d. However, the observed change was statistically significant (*P<* 0.05) only within the observation group, both in comparison to the 0d and in comparison to the control group. Furthermore, this difference was found to be statistically significant when comparing the two groups (*P<* 0.01) (**Table 3**).

**Table 3:** Comparison of changes in blood glucose-related indexes before and after observation between the observation group and the control group ( $\bar{X\;}$ ± s).

Parameters	Observation time point (d)	Observation group	Control group	*t*	*P* value
FPG (mmol/L)	0	8.96 ± 1.91	10.08 ± 2.96	1.691	0.097
	14	7.25 ± 1.05*^†^	7.92 ± 1.67*	1.794	0.079
	28	5.99 ± 0.74*^†^	7.98 ± 1.64*	5.972	0.000^#^
2hPG (mmol/L)	0	13.10 ± 2.83	13.12 ± 4.44	0.026	0.980
	14	10.05 ± 1.97*^†^	10.46 ± 2.32*	0.699	0.488
	28	7.69 ± 1.30*^†^	10.37 ± 2.11*	5.710	0.000^#^
eHbA1c (%)	0	8.79 ± 1.82	9.31 ± 2.10	0.943	0.350
	14	6.65 ± 0.96*^†^	8.01 ± 1.76*	3.616	0.001^#^
	28	6.02 ± 0.60*^†^	8.49 ± 1.96	6.616	0.000^#^

*P< 0.05 compared with the 0d of own group.

^†^P< 0.05 compared with the 14d of own group.

^#^P< 0.05 compared the observation group with the control group at the 0d, 14d, 28d.

Fasting blood glucose (FPG), 2-hour postprandial blood glucose (2hPG), estimated glycated hemoglobin (eHbA1c).

### 3.2 Comparison of changes in CGM monitoring indicators before and after observation

#### 3.2.1 Changes in blood glucose fluctuations index

**Figure 2:**
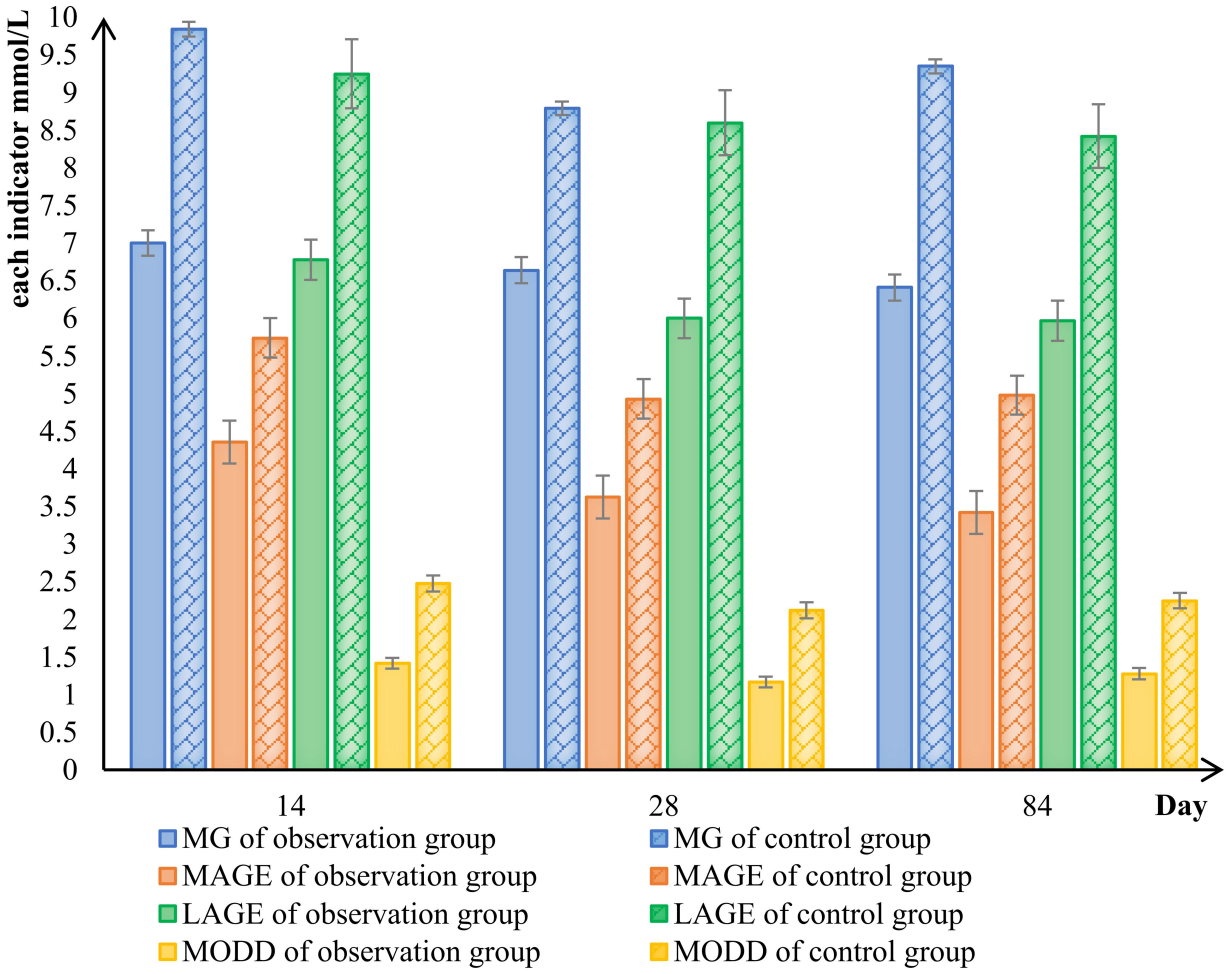
Comparison of changes in blood glucose fluctuation indicators between the observed and control groups. mean blood glucose (MG), large amplitude of glycemic excursions (LAGE), mean amplitude of glycemic excursions (MAGE), mean of daily differences (MODD).

The results indicated that after the 28d of treatment with the Qingre Lishi decoction, the SD, CV, LAGE, MAGE, and MODD of patients in the observation group exhibited a decreasing trend (**Figure 2**). The differences in LAGE, MAGE, and MODD were statistically significant (*P<* 0.01) when compared to both the 0d and the control group (**Table 4**).

**Table 4:** Comparison of changes in CGM indexes before and after observation between the observation group and the control group [ $\bar{X\;}$ ± s or M (Ql, Qu)].

Parameters	Observation time point (d)	Observation group	*P* value	Control group	*P* value	*t*/*Z*	*P* value
TIR (%)	1	76.60 (69.90,87.40)		76.85 (66.00,85.25)		-0.036	0.971
	14	91.80 (85.30,96.80)	0.000	77.35 (66.00,83.35)	0.249	-4.008	0.000^#^
	28	94.60 (88.70,99.00)	0.001*	66.95 (41.48,79.35)	0.020^†^	-5.335	0.000^#^
TAR (%)	1	19.20 (10.30,25.30)		21.55 (10.75,23.45)		-0.081	0.935
	14	8.00 (2.10,12.90)	0.000	21.55 (14.40,23.68)	0.400	-4.026	0.000^#^
	28	4.10 (0.90,10.00)	0.003*	32.70 (20.50,58.38)	0.007^†^	-5.335	0.000^#^
TBR (%)	1	2.70 (0.40,4.90)		1.60 (0.25,4.08)		-0.859	0.416
	14	1.20 (0.20,1.80)	0.003*	1.45 (0.10,3.68)	0.917	-1.647	0.390
	28	0.70 (0.10,1.80)	0.191	0.30 (0.00,1.43)	0.032^†^	-0.826	0.409
MG (mmol/L)	1	7.35 ± 1.68		7.71 ± 1.74		-0.762	0.449
	14	7.00 ± 1.36	0.074	7.66 ± 1.52	0.593	-1.653	0.104
	28	6.64 ± 1.00	0.000*	8.79 ± 1.79	0.013^†^	-5.230	0.000^#^
CV (%)	1	24.72 (21.75,27.11)		22.39 (20.67,25.86)		-0.993	0.321
	14	23.24 (21.23,27.19)	0.737	27.30 (22.99,28.49)	1.000	-0.478	0.632
	28	21.67 (19.16,24.78)	0.063	24.41 (21.41,31.18)	0.355	-2.175	0.030^#^
LAGE (mmol/L)	1	7.11 ± 1.53		6.97 ± 1.71		0.308	0.760
	14	6.78 ± 1.07	0.265	7.08 ± 1.66	0.113	-0.798	0.429
	28	6.00 ± 1.51	0.000*	8.60 ± 2.11	0.245	-5.230	0.000^#^
MAGE (mmol/L)	1	4.13 ± 1.02		3.99 ± 1.10		0.449	0.655
	14	4.36 ± 1.18	0.276	4.00 ± 1.11	0.476	-3.691	0.152
	28	3.63 ± 1.02	0.001*	4.93 ± 1.65	0.035^†^	-3.533	0.001^#^
MODD (mmol/L)	1	1.56 (1.28,2.11)		1.66 ± 0.66		-0.045	0.964
	14	1.42 (1.27,1.55)	0.134	1.67 ± 0.65	0.198	-5.411	0.299
	28	1.17 (0.97,1.49)	0.007*	2.12 ± 0.65	0.112	-5.814	0.000^#^

*P< 0.05 compared with the 0d of own group.

^†^P< 0.05 compared with the 14d of own group.

^#^P< 0.05 compared the observation group with the control group at the 0d, 14d, 28d.

Time in range (TIR), time above range (TAR), time below range (TBR), mean blood glucose (MG), coefficient of variation (CV), large amplitude of glycemic excursions (LAGE), mean amplitude of glycemic excursions (MAGE), mean of daily differences (MODD).

In pairwise analysis within the observation group, it was observed that compared to the 14d, the amplitude of blood glucose fluctuation was significantly improved after the patients were treated with Qingre Lishi decoction. And the changes in LAGE, MAGE, and MODD were statistically significant (*P<* 0.05). However, in the control group, which underwent only lifestyle intervention, it was found that changes in LAGE, MAGE, and MODD were not significant, and the changes in SD and CV exhibited an increasing trend. Additionally, the upward change in SD was statistically significant compared to the 0d (*P<* 0.05). This indicates that patients who only underwent lifestyle interventions had greater fluctuations in their own blood glucose (**Table 4**).

#### 3.2.2 Changes in blood glucose compliance index

Paired analyses showed statistically significant differences in TIR, TAR, and TBR compared to the 0d among patients in the observation group (*P<* 0.05). Furthermore, when comparing the 28d to the 14d with the Qingre Lishi decoction, significant differences in TIR and TAR were observed (*P<* 0.01). Meanwhile, we noted slight improvements in TIR and MG through lifestyle intervention alone, but after the 28d of intervention, we observed a rebound trend in TIR, TAR, TBR, and MG among the patients (**Table 4**).

**Figure 3:**
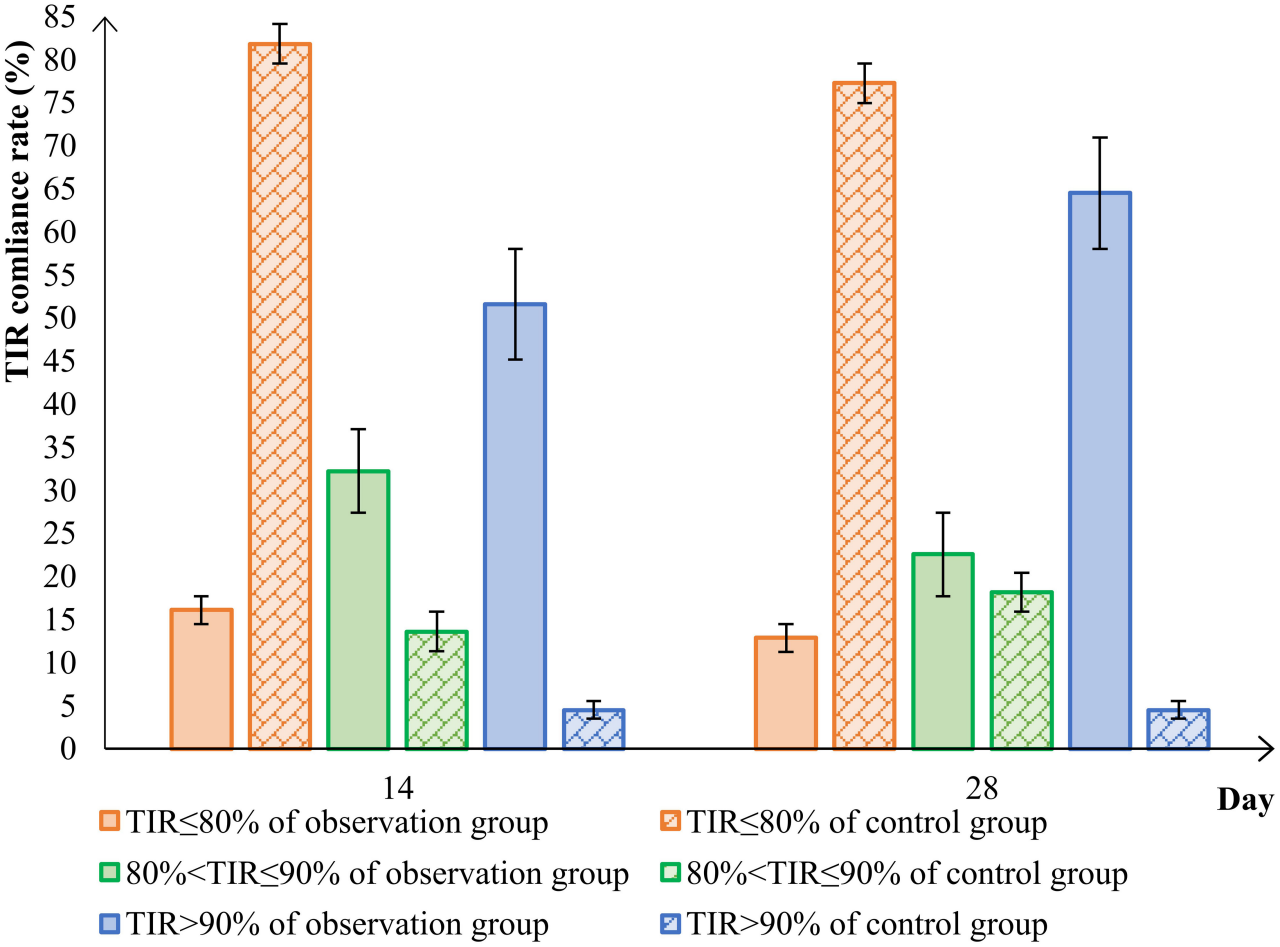
Comparison of TIR attainment in the observation group and control group. time in range (TIR).

In conclusion, using TIR > 80% as the measure of success, the TIR attainment rate in the observation group significantly improved to 87.10% after the 28d of treatment with the Qingre Lishi decoction. This indicates a notable upward trend in TIR and significant relief in blood glucose levels among the patients. On the other hand, the TIR attainment rate in the control group was only 22.73%. Moreover, as time progressed, the changes in TIR, TAR, TBR, and MG were not evident and even exhibited a rebound effect (**Table 5**; **Figure 3**).

**Table 5:** Comparison of TIR attainment at each observation stage between the observation group and the control group.

Parameters	Observation time point (d)	Observation group	Control group
TIR ≤ 80%	14	5 (16.12)	18 (81.82)
	28	4 (12.90)	17 (77.27)
80%< TIR ≤ 90%	14	10 (32.26)	3 (13.64)
	28	7 (22.58)	4 (18.19)
TIR > 90%	14	16 (51.62)	1 (4.54)
	28	20 (64.52)	1 (4.54)

Time in range (TIR).

#### 3.2.3 Changes in AGP mapping

**Figure 4:**
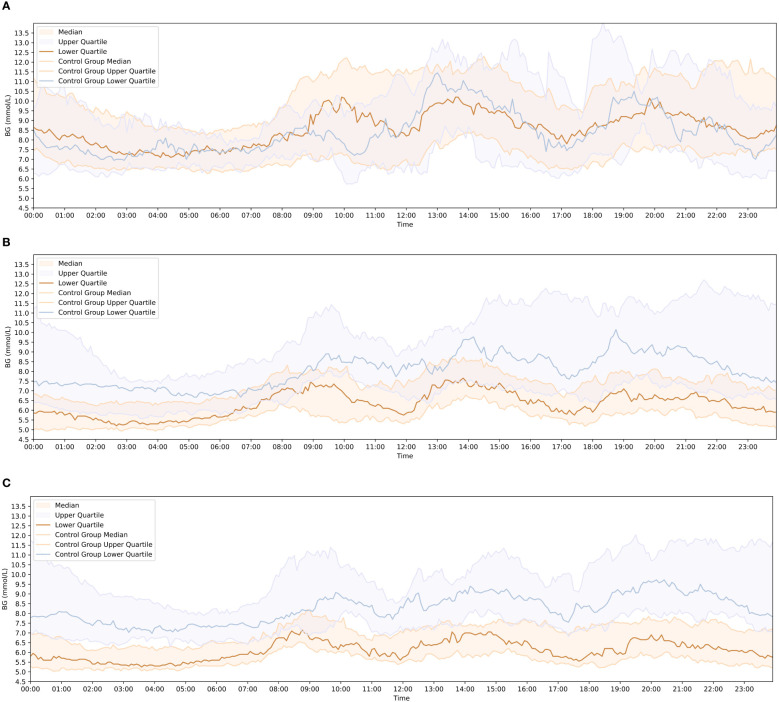
Comparison of blood glucose change curves between the observation and control groups at each observation time point. **(A)** Blood glucose fluctuation profiles of patients in the observation group and control group on the 1 day of wearing CGM. **(B)** Blood glucose fluctuation profiles of patients in the observation group and control group on the 14 day of wearing CGM. **(C)** Blood glucose fluctuation profiles of patients in the observation group and control group on the 28 day of wearing CGM.

The 1d, 14d, and 28d after wearing CGM were chosen as observation points. The matplotlib library for Python used to visualize AGP in both groups of patients. The results showed that before the 0d, the majority of newly diagnosed T2DM patients in the group had high blood sugar levels and experienced significant fluctuations. Their hyperglycemic condition improved after lifestyle intervention or treatment with the addition of the Qingre Lishi decoction (**Figure 4**).

At the 14d of observation, there were already noticeable differences in AGP maps between the two groups of patients. At the 28d of observation, the patients in the observation group exhibited more stable AGP maps and experienced less fluctuation in blood glucose levels compared to the control group. The observation group showed improvements in hyperglycemia, with reduced and stabilized fluctuations in the blood glucose change curve compared to the previous one. After 28d of clinical treatment, the blood glucose change curve in the observation group became smoother, with smaller fluctuation amplitudes, which was significantly improved compared with the 0d (**Figure 4**).

### 3.3 Adverse reactions

We observed that in the observation group, all newly diagnosed overweight and obese T2DM patients did not exhibit significant abnormalities in ALT, AST, GGT, UREN, and Scr after treatment with the Qingre Lishi decoction. The differences in the changes were not statistically significant (*P* > 0.05). It means that the Qingre Lishi decoction will not affect liver and kidney functions in humans. In terms of hypoglycemic events, there were 2 cases of hypoglycemia in the observation group and 6 cases of hypoglycemia in the control group, the incidence of hypoglycemia in the two groups was 6.45% and 27.27% respectively, demonstrating a statistically significant difference in the occurrence of hypoglycemia according to the chi-square test (*P<* 0.05) (**Table 6**).

**Table 6:** Comparison of the incidence of hypoglycemia between the observation group and the control group.

Parameters	Observation group	Control group	*χ* ^2^	*P* value
Hypoglycemia[*n* (%)]	2 (6.45)	6 (27.27)	4.353	0.037

## 4 Discussion

The results of this study suggested that treatment with the Qingre Lishi decoction could significantly relieve blood glucose levels in overweight and obese patients with newly diagnosed T2DM. It was found that from the 14th day of observation, the changes in eHbA1c of the patients in the observation group were statistically significant when compared with the control group. At the 28d, the changes in FPG and 2hPG of the patients in the observation group started to show statistical differences. We believe that this may be related to the fact that Chinese herbal tonics require a certain amount of time to accumulate efficacy in controlling blood glucose and regulating the patient’s internal environment stability. This finding was also consistent with the results described in a previous study by Liying Zhang (32). Comparison within the group revealed that in those who adhered to the Qingre Lishi decoction for 28 days, the patients’ FPG, 2hPG and eHbA1c were significantly relieved, and their quality of life was improved. It also provided a good foundation for remission of newly diagnosed T2DM. This was consistent with the findings of the latest real-world study by Prof. Guoming Pang’s team (33, 34).

Another strength of our study was the use of a continuous glucose monitoring system in order to demonstrate that the Qingre Lishi decoction reduces glycemic fluctuations and enhances glycemic stability in newly diagnosed overweight and obese T2DM patients, with a low incidence of hypoglycemia and a safety profile. Glycemic fluctuations have now been shown to increase vascular endothelial oxidative stress, thus becoming an independent risk factor for vascular complications in diabetes. Our team’s previous study has also demonstrated that IL-6 levels are significantly elevated in newly diagnosed T2DM patients of the type of dampness heat trapped spleen, and that oxidative stress damage to the vascular endothelium is more severe (18). Meanwhile, it has also been shown in a large number of studies that beneficial genera such as rumenococci and Bradyrhizobium spp are diminished in the intestinal flora of obese T2DM patients. This reduction weakens bile acid metabolism, resulting in glucose metabolism disorders, decreased insulin sensitivity, and significant fluctuations in blood glucose levels (21, 22, 35, 36). Therefore, the focus of clinicians has shifted towards achieving a more precise and effective reduction in blood glucose levels while also minimizing fluctuations.

In recent years, there has been an increasing number of studies using CGM to evaluate TIR in patients with T2DM. Moreover, TIR has been proposed as a valuable addition to the glycemic control targets (9). After evaluating CGM data, we observed that as the duration of the treatment with the Qingre Lishi decoction increased, patients achieved higher rates of TIR. Additionally, TAR decreased significantly, while the change in TBR was not conspicuous. After the 28d of treatment with the formula, patients exhibited smoother blood glucose change patterns within a 24-hour period, and the amplitude of blood glucose fluctuation was significantly reduced. To assess blood glucose variability, we used SD and CV, and then, we found that after treatment with the Qingre Lishi decoction, the patients’ blood glucose fluctuations decreased compared to the 0d. On the other hand, relying solely on lifestyle interventions did not relieve blood glucose fluctuations and, in fact, tended to exacerbate their intensity. This further suggests that the administration of the Qingre Lishi decoction can effectively decrease blood glucose fluctuations and strengthen blood glucose regulation. This study also discovered that in the short term, lifestyle interventions alone could partially manage blood glucose, though to a limited extent. However, in the long run, the majority of patients struggle to maintain a consistent and appropriate diet and exercise routine. As a consequence, glycemic control rates diminish, blood glucose experiences significant fluctuations, and then, in some cases, a rebound effect may manifest.

The incidence of newly diagnosed T2DM in China is gradually rising. It is marked by a substantial number of individuals being overweight or obese, low adherence to glycemic control, and insufficient patient awareness. However, TCM treatment for T2DM possesses distinct advantages and has garnered increasing recognition. In recent years, large-scale clinical trials have confirmed that TCM has made good achieved positive outcomes in regulating overall metabolic functions and managing blood glucose levels (13, 37, 38). These findings open up new possibilities for TCM to effectively manage blood glucose levels in newly diagnosed T2DM patients, aiming for stable blood sugar levels and achieving remission of newly diagnosed T2DM. However, it is important to note that there is limited research on the use of herbal formulas alone as interventions for controlling blood glucose in newly diagnosed T2DM patients. Therefore, we combined the CGM technique to confirm the effectiveness and safety of herbal formula in mitigating and stabilizing blood glucose in newly diagnosed T2DM patients from the perspective of clinical medical research.

In this study, the selected drugs were based on Da Chai Hu Decoction as the fundamental formula. Modern pharmacological studies have confirmed that the active ingredients in the drugs of the formula possess anti-inflammatory properties, prevent vascular endothelial oxidation, lower blood glucose levels, and improve insulin resistance. For example, the Chaihu polysaccharides and Chaihu saponins in radix bupleuri (Chai Hu, 柴胡) can increase the sensitivity to inhibit inflammatory signaling pathways, such as the HMGB1-TLR4 signaling pathway, inhibit oxidative stress, and activate the 5-HT2C receptor that suppresses appetite in humans. Thus they play a crucial role in preventing inflammation, inhibiting the oxidation of the vascular endothelium, and preventing weight gain (39–41). Scutellaria baicalensis (Huang Qin, 黄芩) decoction regulates the metabolism of substances in the body thereby reducing blood lipids and treating obesity; it inhibits NO production, exerts anti-inflammatory effects, and is closely related to diseases such as edema, hypertension and heart disease (42, 43). The organic acids and polysaccharides contained in dark plum (Wu Mei, 乌梅) can exhibit antioxidant activity, protect pancreatic β-cells, and improve insulin resistance, thereby reducing blood glucose levels. The organic acids present in dark plum (Wu Mei, 乌梅) can also reduce oxidative stress and inflammation by modulating the Nrf2/ARE signaling pathway and inhibiting ROS overproduction (44, 45). Additionally, a large number of animal experiments have also confirmed the effectiveness of the drugs in the formula (46, 47). For instance, rhizoma zingiberis (Gan Jiang, 干姜) decoction can resist vascular oxidation, inhibit platelet aggregation, improve lipid metabolism, and also improve cardiac function by modulating Ang II, TNF-α, MDA, and NO (48–50). In both *in vivo* and *in vitro* studies, cinnamaldehyde in cassia twig (Gui Zhi, 桂枝) has been shown to be the component most closely associated with reducing blood glucose levels (51–53). The team from Iran even demonstrated that aqueous extract of rhubarb (Da Huang, 大黄) had a positive effect on insulin resistance and lipoproteins in T2DM patients (54).

As far as we know, the strength of this study lies in its clinical approach, combining CGM monitoring techniques with the use of herbal formulas to control glycemia in newly diagnosed overweight and obese T2DM. However, there are some limitations and shortcomings in this study. Firstly, it is limited by a small sample size and a short follow-up period. Secondly, when it comes to acquiring observational metrics, we only compared the differences between pre- and post-observations of eHbA1c in the enrolled patients, disregarding the differences in HbA1c itself. Additionally, we only collected the fasting C-peptide values at the time of enrollment and did not collect their fasting insulin values, which prevented us from calculating the HOMA-β and Matsuda index to assess the effect of the Qingre Lishi decoction on the improvement of insulin resistance and insulin secretion/sensitivity in the patients. Thirdly, although each patient received formal diabetic diet and exercise instructions, their related behaviors were not meticulously recorded, which could also have an impact on blood glucose indicators.

In the future, we plan to conduct a large-sample, multi-center randomized controlled trial to validate the effectiveness of the Qingre Lishi decoction in managing blood glucose, improving insulin resistance and alleviating blood glucose fluctuations among individuals newly diagnosed with T2DM from multiple perspectives.

## 5 Conclusion

The Qingre Lishi decoction can effectively improve the hyperglycemic condition of overweight and obese patients with newly diagnosed T2DM. It can reduce blood glucose, alleviate blood glucose fluctuations, reduce the incidence of hypoglycemia, and improve patients’ adherence and self-confidence in controlling their own blood glucose.

## Data availability statement

The raw data supporting the conclusions of this article will be made available by the authors, without undue reservation.

## Ethics statement

The studies involving humans were approved by Ethics Committee of the Affiliated Hospital of Liaoning University of Traditional Chinese Medicine Liaoning University of Traditional Chinese Medicine. The studies were conducted in accordance with the local legislation and institutional requirements. The participants provided their written informed consent to participate in this study. Written informed consent was obtained from the individual(s) for the publication of any potentially identifiable images or data included in this article.

## Author contributions

BW: Writing – original draft, Writing – review & editing. TG: Project administration, Writing – review & editing. ML: Funding acquisition, Supervision, Writing – review & editing. XT: Data curation, Writing – review & editing. JW: Data curation, Writing – review & editing.
